# Early Patient-Centered Outcomes Research Experience With the Use of Telehealth to Address Disparities: Scoping Review

**DOI:** 10.2196/28503

**Published:** 2021-12-07

**Authors:** James E Bailey, Cathy Gurgol, Eric Pan, Shirilyn Njie, Susan Emmett, Justin Gatwood, Lynne Gauthier, Lisa G Rosas, Shannon M Kearney, Samantha Kleindienst Robler, Raymona H Lawrence, Karen L Margolis, Ifeyinwa Osunkwo, Denise Wilfley, Vallabh O Shah

**Affiliations:** 1 Tennessee Population Health Consortium University of Tennessee Health Science Center Memphis, TN United States; 2 Patient-Centered Outcomes Research Institute Washington, DC United States; 3 Westat Inc Center for Healthcare Delivery Research and Evaluation Rockville, MD United States; 4 Department of Head and Neck Surgery and Communication Sciences Duke University School of Medicine Duke Global Health Institute Durham, NC United States; 5 College of Pharmacy University of Tennessee Health Science Center Memphis, TN United States; 6 Department of Physical Therapy and Kinesiology Zuckerberg College of Health Sciences University of Massachusetts Lowell, MA United States; 7 Department of Epidemiology and Population Health Division of Primary Care and Population Health Stanford School of Medicine Palo Alto, CA United States; 8 Department of Medicine Division of Primary Care and Population Health Stanford School of Medicine Palo Alto, CA United States; 9 Solution Insights & Validation Highmark Health Pittsburgh, PA United States; 10 Norton Sound Health Corporation Nome, AK United States; 11 Community Health Behavior and Education Jiann-Ping College of Public Health Georgia Southern University Statesboro, GA United States; 12 HealthPartners Institute Minneapolis, MN United States; 13 Cancer Care Levine Cancer Institute Atrium Health Charlotte, NC United States; 14 Department of Psychiatry College of Medicine Washington University in St. Louis St Louis, MO United States; 15 Department of Internal Medicine and Biochemistry School of Medicine University of New Mexico Albuquerque, NM United States

**Keywords:** telehealth, scoping review, disparities, implementation science

## Abstract

**Background:**

Health systems and providers across America are increasingly employing telehealth technologies to better serve medically underserved low-income, minority, and rural populations at the highest risk for health disparities. The Patient-Centered Outcomes Research Institute (PCORI) has invested US $386 million in comparative effectiveness research in telehealth, yet little is known about the key early lessons garnered from this research regarding the best practices in using telehealth to address disparities.

**Objective:**

This paper describes preliminary lessons from the body of research using study findings and case studies drawn from PCORI seminal patient-centered outcomes research (PCOR) initiatives. The primary purpose was to identify common barriers and facilitators to implementing telehealth technologies in populations at risk for disparities.

**Methods:**

A systematic scoping review of telehealth studies addressing disparities was performed. It was guided by the Arksey and O’Malley Scoping Review Framework and focused on PCORI’s active portfolio of telehealth studies and key PCOR identified by study investigators. We drew on this broad literature using illustrative examples from early PCOR experience and published literature to assess barriers and facilitators to implementing telehealth in populations at risk for disparities, using the active implementation framework to extract data. Major themes regarding how telehealth interventions can overcome barriers to telehealth adoption and implementation were identified through this review using an iterative Delphi process to achieve consensus among the PCORI investigators participating in the study.

**Results:**

PCORI has funded 89 comparative effectiveness studies in telehealth, of which 41 assessed the use of telehealth to improve outcomes for populations at risk for health disparities. These 41 studies employed various overlapping modalities including mobile devices (29/41, 71%), web-based interventions (30/41, 73%), real-time videoconferencing (15/41, 37%), remote patient monitoring (8/41, 20%), and store-and-forward (ie, asynchronous electronic transmission) interventions (4/41, 10%). The studies targeted one or more of PCORI’s priority populations, including racial and ethnic minorities (31/41, 41%), people living in rural areas, and those with low income/low socioeconomic status, low health literacy, or disabilities. Major themes identified across these studies included the importance of patient-centered design, cultural tailoring of telehealth solutions, delivering telehealth through trusted intermediaries, partnering with payers to expand telehealth reimbursement, and ensuring confidential sharing of private information.

**Conclusions:**

Early PCOR evidence suggests that the most effective health system- and provider-level telehealth implementation solutions to address disparities employ patient-centered and culturally tailored telehealth solutions whose development is actively guided by the patients themselves to meet the needs of specific communities and populations. Further, this evidence shows that the best practices in telehealth implementation include delivery of telehealth through trusted intermediaries, close partnership with payers to facilitate reimbursement and sustainability, and safeguards to ensure patient-guided confidential sharing of personal health information.

## Introduction

### Background

People living in medically underserved, low-income, or rural areas; those from racial and ethnic minorities; members of the lesbian, gay, bisexual, and transgender (LGBT) community; and those with limited English proficiency or disabilities often face substantial barriers to accessing needed health care and are at high risk for health disparities [1,2]. About one-quarter of the US population experiences geographic and/or economic disparities. For example, 34 million Americans (10.5%) live in poverty [3] and some 46 million Americans (15%) live in rural areas [4]. These populations are more likely to report issues with access to primary and specialty care, longer travel time to providers, lower rates of insurance coverage, and higher rates of chronic conditions than urban resident [2,5-7]. Racial and ethnic disparities are also common and are strongly linked to social determinants of health [1,5,8].

Other populations at particularly high risk for disparities include low-income African Americans in medically underserved urban and rural areas of the South [9-11], American Indian populations in rural areas [8,12,13], the mostly Alaskan Native residents of Alaska [14], and Latinx Americans, who face language, insurance, and other barriers to access to care [8]. Additional groups identified by the Patient-Centered Outcomes Research Institute (PCORI) as priority populations include LGBT persons, those with low health literacy/numeracy and limited English proficiency, and those with disabilities [15-21]. All these populations face barriers related to the social determinants of health that prevent adequate access to care [13,19-23], resulting in poor health outcomes across a multitude of domains.

### Potential for Using Telehealth to Address Disparities

Telehealth modalities have great potential to help overcome geographic, socioeconomic, cultural, and language barriers related to the social determinants of health and enhance access to essential health services for high-risk populations [24,25]. Telehealth has traditionally been a way to provide health care access in rural communities, and its adoption has accelerated for these populations during the COVID-19 pandemic [26,27]. While the potential for telehealth to address disparities, such as transportation and availability of providers, is high, preliminary data during the COVID-19 pandemic suggest that differences in internet and telehealth access may actually compound disparities in chronic disease outcomes [28]. Telehealth can be used for multiple purposes, including direct delivery of care to patients and their caregivers, remote monitoring of health outcomes, education of patients and caregivers, support for self-care and health behavior change, assistance with health care decision-making, and communication of test results [29]. Likewise, telehealth can employ multiple communication modalities, including telephone, video teleconference, text messaging, mobile apps, and wearable monitors, that allow providers to remotely communicate, monitor, and share information with patients [30-32]. Previous research has documented the effectiveness of these telehealth modalities in reaching rural populations to provide care, prescreening evaluations, and patient education [25,33,34], yet data on the effectiveness of telehealth in other populations is more limited [29-32]. Moreover, while many health care delivery systems have implemented telehealth solutions to address barriers to access and better serve populations at risk for disparities, outcomes and sustainability have been variable [24,25,35].

### Study Aims

The primary purpose of this scoping review was to identify key barriers to telehealth implementation and describe how barriers can be addressed in populations at risk for disparities. PCORI has invested US $386 million in comparative effectiveness research in telehealth, yet little is known about the key early lessons of this research for using telehealth to address disparities. This review describes preliminary lessons from early patient-centered outcomes research (PCOR) experience and literature, using study findings and illustrative case studies drawn from these seminal PCOR initiatives and literature.

## Methods

### Overview

A systematic scoping review of telehealth studies addressing disparities was performed and was guided by the Arksey and O’Malley Scoping Review Framework [36], with a focus on PCORI’s active portfolio of telehealth studies and key supporting PCOR identified by several of the investigators.

### Identifying the Research Question

The purpose of this scoping review was to describe barriers and facilitators to implementing telehealth or remote interventions in populations at risk of health disparities (eg, racial and ethnic minority groups or people living in rural areas) within comparative effectiveness research projects funded by PCORI. The specific question addressed was as follows: “How have PCORI-funded investigators overcome barriers to implementing telehealth interventions in populations at risk for disparities?”

### Identifying Relevant Studies

Between December 2012 and March 2019, PCORI funded 84 comparative effective research projects, in which at least one of the study’s comparison arms used telehealth to improve health outcomes. PCORI defines telehealth as the delivery of health services via remote telecommunication modalities, such as telephonic communication, remote monitoring devices, real-time videoconferencing, and mobile devices. For this scoping review, PCORI staff first reviewed the telehealth portfolio to identify studies aiming to improve health outcomes for populations at risk of or facing disparities. Studies included those that targeted at least one of the following PCORI priority populations at risk for disparities: people living in medically underserved, low-income, or rural areas; those representing racial and ethnic minorities; members of the LGBT community; those with low health literacy/numeracy and limited English proficiency; and those with disabilities [37]. Other PCORI populations of interest, including older adults, women, children, individuals with multiple chronic diseases, individuals with rare diseases, individuals whose genetic makeup affects their medical outcomes, veterans, and members of the armed forces and their families, were not specifically included in this scoping review.

Of the 84 PCORI-funded studies in this portfolio, PCORI staff identified 41 studies focused on improving health outcomes for populations at risk of or facing disparities by confirming the target population for each study. Qualifying studies were further reviewed by PCORI staff to determine if any barriers to implementation of the planned telehealth intervention were experienced and whether the investigators were able to mitigate these barriers to successfully implement the study telehealth intervention. A convenience sample of 8 studies was selected based on their representativeness (ie, to ensure that all major targeted populations at risk for disparities were included), study phase (ie, studies either completed or nearing completion), and perceived principal investigator availability to provide illustrative examples of successful approaches for working through obstacles in implementing telehealth interventions for diverse populations.

### Identifying Barriers and Facilitators

We drew on these 8 illustrative examples from early PCOR experience and evidence from the literature to assess barriers and facilitators to implementing telehealth in populations at risk for disparities [25]. Key informant interviews of principal investigators were conducted for the convenience sample of 8 PCORI studies to identify common challenges in the implementation of telehealth in populations at risk for disparities. Among the 8 studies in the convenience sample, 6 principal investigators were contacted for phone interviews and 4 interviews were completed. PCORI then hosted a webinar attended by investigators from all 8 of the studies to facilitate discussion about the study challenges investigators faced and the solutions they identified and implemented. After the initial information gathering from the interviews and webinar, PCORI staff invited investigators to participate in this scoping review and share the lessons learned.

### Extracting the Data

**Figure 1:**
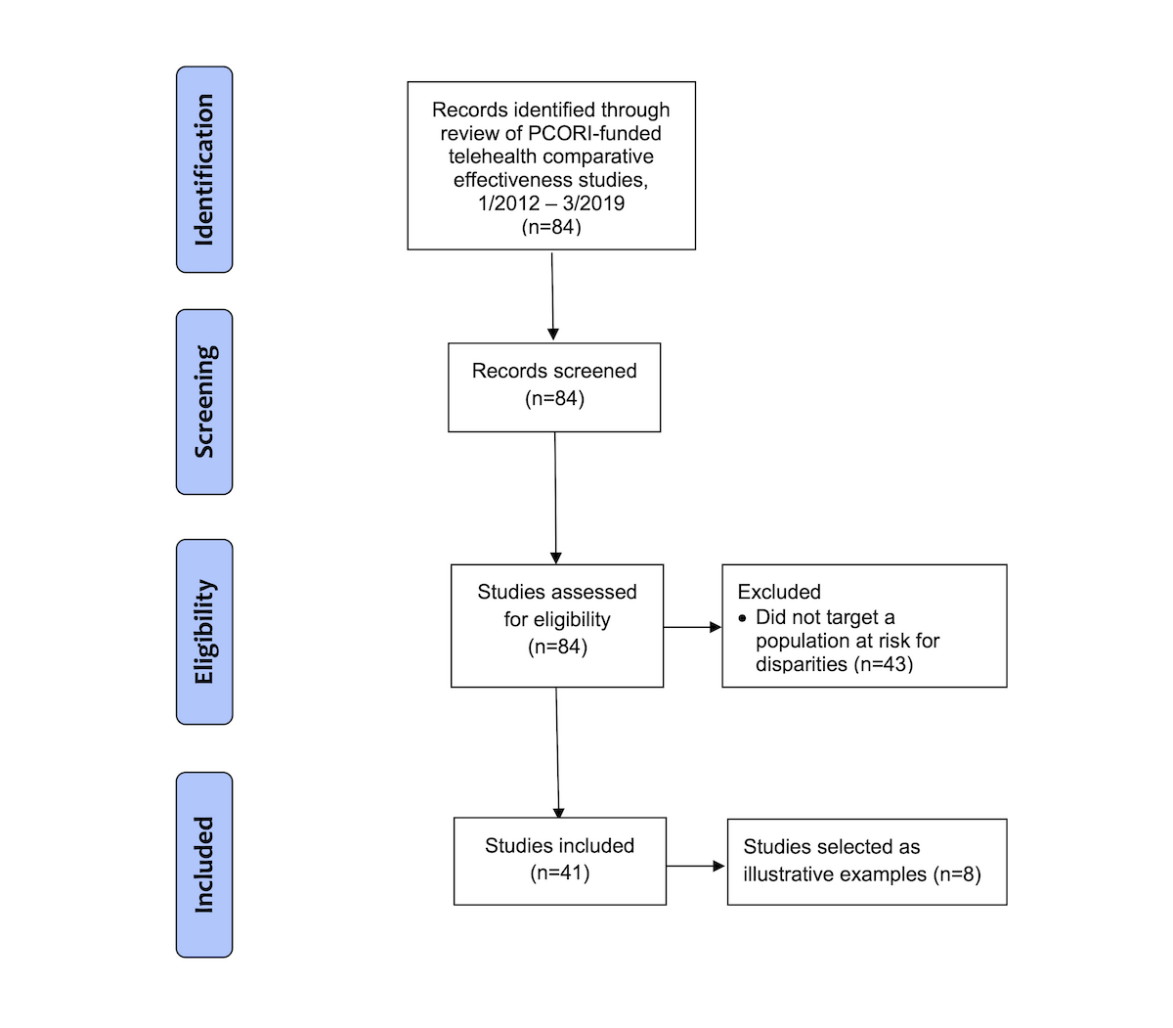
PRISMA (Preferred Reporting Items for Systematic Reviews and Meta-Analyses) extension for scoping reviews flow diagram of the study selection process. PCORI: Patient-Centered Outcomes Research Institute.

Following identification of all PCORI studies meeting the criteria for the scoping review (n=41), PCORI staff extracted descriptive data for all these studies, including telehealth purpose and modality, population, health condition, budget amount, principal investigator, and institution, using published project descriptions and study papers. PCORI staff and study investigators then extracted additional data from these same sources and interim progress reports (where available) in order to categorize documented barriers and facilitators using a published framework of identified barriers (eg, limitations) and facilitators (eg, solutions) to telehealth [25]. We then used prespecified data definitions from the Fixsen active implementation framework to further categorize barriers and facilitators to implementing telehealth within 3 major domains, including competency drivers (ie, participant selection, training, and supervision), organizational drivers (ie, decision support, administrative support, and system intervention), and leadership drivers [38,39]. The reporting process used the PRISMA (Preferred Reporting Items for Systematic Reviews and Meta-Analyses) extension for scoping reviews (Figure 1) [40,41].

### Collating, Summarizing, and Reporting the Data

A table including the core descriptive data regarding all PCORI-funded telehealth studies addressing populations at risk for disparities was created. In addition, figures and charts were created to summarize project details about this subset of studies. An assessment of the quality of studies included in this review was not performed. A table was created summarizing the barriers and facilitators identified within each major implementation domain. Finally, major themes regarding how telehealth interventions can overcome barriers to telehealth adoption and implementation were identified through review of both the extracted data and illustrative examples using an iterative Delphi process among the 8 PCORI principal investigators participating in the study to achieve consensus. The Delphi process was facilitated by PCORI staff and a lead principal investigator for the scoping review, and was conducted through teleconference and email communication. PCORI staff and the lead principal investigator initially categorized extracted data and illustrative examples by barrier and facilitator types based on previously published categories [25]. Studies were then recategorized and category labels were revised based on feedback from participating investigators until consensus on the barrier and facilitator types was achieved.

## Results

### Identifying Relevant Studies

**Figure 2:**
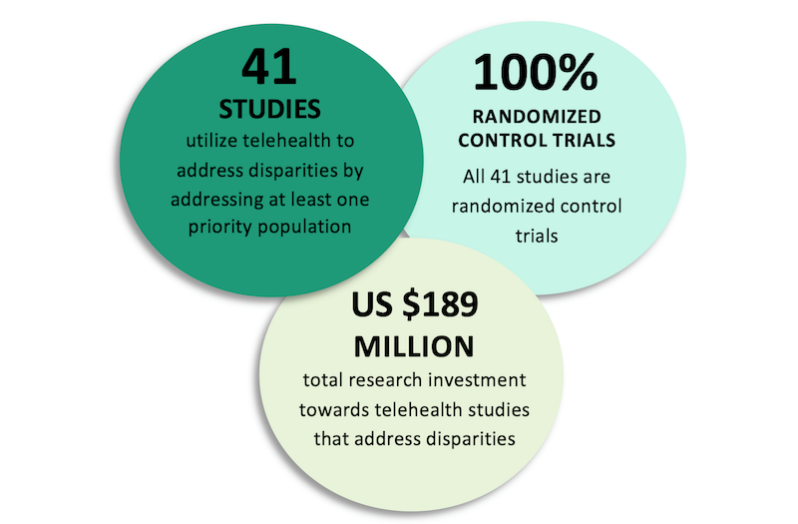
Patient-Centered Outcomes Research Institute comparative effectiveness studies assessing telehealth solutions to address disparaties.

As shown in Figure 2, 41 PCORI-funded studies were identified that assessed the use of telehealth to improve outcomes for populations at risk for health or health care disparities in this analysis. Of note, all 41 studies employed a randomized controlled trial methodology, and all were pragmatic or “real-world” comparative effectiveness studies rather than efficacy studies.

### Study Characteristics

The descriptions, study goals, telehealth interventions studied, settings, number of participants, and targeted study populations for these 41 PCORI-funded studies are detailed in Multimedia Appendix 1 [42-96]. The studies ranged in size from 59 to 84,828 participants; all were conducted in ambulatory settings; and the majority (33/41, 80.5%) were conducted in primary care and/or community settings.

**Figure 3:**
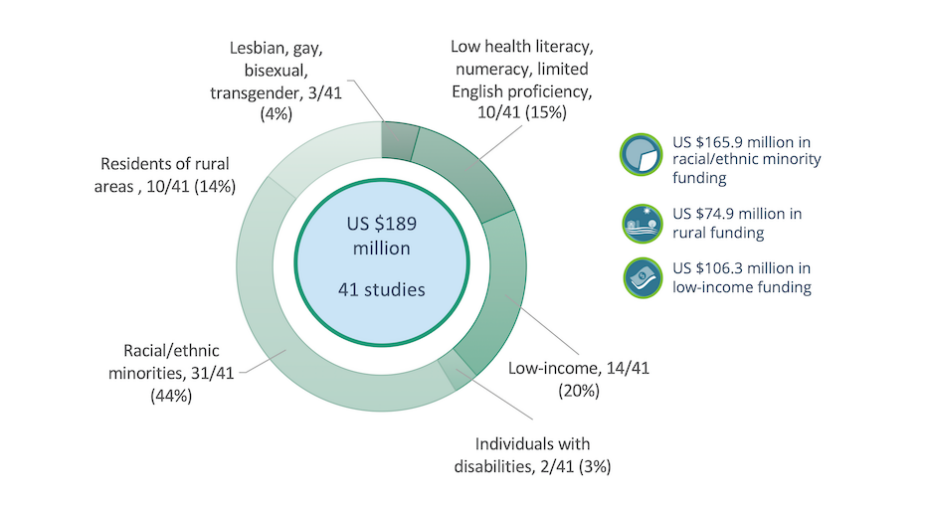
Populations at risk of disparities targeted by Patient-Centered Outcomes Institute comparative effectiveness studies in telehealth.

As shown in Figure 3, all 41 studies targeted at least one of PCORI’s priority populations, including racial and ethnic minorities, people living in rural areas, people with low income or low socioeconomic status, people with low health literacy, members of the LGBT community, and people with disabilities.

PCORI-funded studies employed multiple telehealth modalities. The majority of these 41 studies used a mobile device (n=29, 71%) or were web-based (n=30, 73%). Fewer studies used real-time videoconferencing (n=15, 37%), remote patient monitoring (n=8, 20%), or store-and-forward capability (ie, asynchronous electronic transmission; n=4, 10%). It is important to note that a similar telehealth modality does not indicate similar studies. For example, studies employing mobile device interventions were diverse and included web-based interventions requiring full internet access, as well as lower-tech interventions that only employed SMS or simple text messaging. All the web-based interventions referenced below required reliable broadband internet access. Real-time videoconferencing was used in 15 studies, and 11 of these studies utilized telehealth to improve access to primary or specialty care. Health conditions involving videoconferencing were cardiovascular diseases (n=2), kidney diseases (n=2), mental/behavioral health (n=4), nutritional and metabolic disorders (n=3), allergies and immune disorders, multiple chronic conditions, neurological disorders, respiratory diseases, and trauma/injury. Remote patient monitoring (eg, blood pressure or blood glucose levels) was used in 8 studies. Remote monitoring varied from a wearable device (eg, Fitbit) to mobile app monitoring to an in-home device (eg, scale, blood pressure monitor, or video monitoring). Health conditions incorporating remote monitoring included cardiovascular diseases (n=3), mental/behavioral health (n=2), nutritional and metabolic disorders, and respiratory diseases. Store-and-forward technology was employed in 5 studies that included the following conditions: functional limitations and disabilities, mental/behavioral health, rare diseases, hearing loss, and skin diseases.

Furthermore, the purpose of the 41 PCORI telehealth interventions also varied substantially, with 8 (20%) focused on enabling health monitoring, 20 (49%) focused on improving access to specialty care, 22 (54%) focused on health education, and 27 (66%) focused on promoting chronic condition self-management. The spectrum of chronic conditions studied also varied substantially. The largest proportion of studies focused on behavioral health (n=9, 22%), and nutritional and metabolic disorders (n=6, 15%), including obesity and diabetes. In addition, PCORI has funded telehealth projects addressing disparities focused on cardiovascular conditions (n=3), chronic kidney disease (CKD; n=2), neurological conditions (n=2), reproductive and perinatal health (n=2), rare diseases (n=2), allergies and immune disorders (n=1), cancer (n=1), hearing loss and ear diseases (n=1), functional limitations and disabilities (n=1), infectious diseases (n=1), multiple chronic conditions (n=1), trauma (n=1), and skin diseases (n=1). Although only 1 study focused on patients with multiple chronic conditions, most of the studies included patients with multiple chronic conditions and some additional studies used such presence as an inclusion criterion.

### Identifying Barriers and Facilitators

Barriers and facilitators of telehealth implementation in PCORI-funded studies using telehealth to address disparities are detailed in Tables 1 and 2. Barriers and facilitators are described for only 35 of the 41 studies as 6 were in the early stages of implementation and lacked sufficient data for evaluation. Review of this data revealed that 20 (57%) studies actively engaged patients to assist investigators in building patient-centered design with active patient participation in each development phase, 13 (37%) studies culturally tailored their telehealth intervention, 4 (11%) studies employed partnership with payers to expand telehealth reimbursement, and 11 (31%) studies delivered telehealth care through or with the assistance of trusted intermediaries. Of the 11 studies, 5 (14%) had clinical support team members available to support their telehealth interventions with a human interaction component.

**Table 1:** Barriers to telehealth adoption identified in Patient Centered Outcomes Research Institute–funded studies using telehealth to address disparities.

Barriers (ie, limitations or challenges)			Barriers identified, n	Example
**Competency drivers**				
	**Participant engagement barriers (eg, social determinants of health presenting barriers to telehealth adoption)**			
		Inadequate access to telecommunication technology	9	Patients with many competing social needs and low levels of social support and resources that may have prevented individuals who would otherwise be eligible and/or interested in study participation (eg, inadequate access to technology). This barrier was removed by adding “access to a phone or iPad” to the inclusion criteria.
		Patient and stakeholder feedback	1	Patient and stakeholder partners were integrated into studies to provide feedback on the usability and feasibility of technology.
	**Staff training barriers**		3	Providers and health care staff may not receive training on technology use or telehealth etiquette. Inadequate communication among patients, multiple providers, primary care providers, and specialists exacerbates the technology divide.
	**Inadequate program supervision/management**		3	Lack of personnel structure to oversee administrators of technology platforms.
**Organizational drivers**				
	**Inadequate decision support**			
		Technology problems	0	Not reported.
	**Inadequate administrative support**			
		Insufficient administrative support staff to deliver or support telehealth solutions	5	Lack of support staff available to help access and utilize the different app platforms.
		Health systems workflow	1	Poor integration of telehealth intervention and existing clinical care workflows.
	**Inadequate clinical support**			
		Insufficient staff to deliver telehealth solutions	5	Lack of clinical support, such as providers or lay health workers, available to assist with the delivery of telehealth platforms.
	**System-level barriers**			
		Legal (eg, inadequate safeguards to ensure patient-guided confidential protected health information sharing, state licensure laws, need for credentialing at multiple sites, and liability concerns)	2	Lack of protection for study participants, specifically those who were undocumented immigrants.
		Financial (eg, limited insurance coverage for telehealth)	5	Inconsistent reimbursement coverage for providers. State-based licensure laws exist.
**Leadership drivers**				
	**Inadequate administrative leadership**		0	Not reported.
	**Inadequate clinical leadership (eg, concerns regarding lower quality patient–physician relationship or poor physician buy-in)**		0	Not reported.
	**Partnership**		1	Partnership with national organizations to help design and implement the intervention.

**Table 2:** Facilitators to telehealth adoption identified in Patient Centered Outcomes Research Institute–funded studies using telehealth to address disparities.

Facilitators (ie, solutions or improvements)			Facilitators identified, n	Example
**Competency drivers**				
	**Improvements in participant engagement (eg, approaches to address social needs and facilitate telehealth adoption)**			
		Provision of access to telecommunication technology	4	Case managers assisted patients with addressing telehealth access barriers.
		Patient-centered design (eg, telehealth solutions) actively guided by patients to meet community needs	20	Most studies engaged patients in the design of the intervention and obtained feedback from the target population.
		Cultural tailoring of telehealth solutions to meet the needs of specific communities and populations	13	Culturally tailored interventions for target populations.
	**Improvements in staff training (eg, training in patient empowerment techniques such as motivational interviewing to facilitate telehealth adoption)**		4	Technology training for providers and community health workers. Help solving workflow issues.
**Organizational drivers**				
	**Decision support solutions**			
		Technology solutions	0	Not reported.
	**Administrative solutions**			
		Strengthening of administrative support staff to support telehealth solution	11	Support staff to provide ongoing support and engagement for technical issues.
		Process evaluation	1	Feasibility assessments to determine the fidelity of the technology.
	**Clinical support team solutions**			
		Strengthening clinical support staff to deliver or support telehealth solutions (eg, delivering telehealth through trusted intermediaries)	5	Support staff, such as nurses, providers, and lay health workers, are available to provide human interaction, which strengthens trust with technology.
	**System-level improvements**			
		Legal (eg, safeguards to ensure patient-guided confidential sharing of protected health information)	2	Reassurance for securing data using appropriate computer technology.
		Financial (eg, partnering with payers to expand insurance coverage for telehealth and telehealth reimbursement)	4	Working with communities to provide coverage for community health aides to help implement technology and to better understand telehealth insurance claims.
		Legislative policies	1	Experience working with state legislature/licensure to help lower barriers to telehealth.
**Leadership drivers**				
	**Improvements in administrative leadership**		0	Not reported.
	**Improvements in clinical leadership (eg, engagement of strong physician champions to lead change)**		0	Not reported.

Major themes identified across these studies included the importance of patient-centered design, cultural tailoring of telehealth solutions, delivering telehealth through trusted intermediaries, partnering with payers to expand telehealth reimbursement, and ensuring confidential sharing of private information.

Illustrative examples of these key themes are reviewed in the sections below, and key system- and policy-level changes needed to address barriers to telehealth adoption and implementation are identified.

#### Patient-Centered Design

Patient engagement is a core component of PCORI studies. Some studies go beyond incorporating patient input and feedback on the research and actively engage patients into the study design and overall design of the intervention.

The VIGoROUS (Video Game Rehabilitation for Outpatient Stroke) study illustrates how user-friendly consumer-driven design serves as a critical component of sustainable telehealth initiatives [70,71]. It employed a video gaming system to deliver therapeutic exercises and track adherence/progress remotely for individuals with motor disability. The gaming system was co-designed by therapists and people with motor disability. Artificial intelligence automatically adjusted the difficulty of the exercises to match the ability of the patient, allowing for a simple user interface and less than 1-minute setup.

Similarly, the study by Williams et al comparing high-touch and high-tech care in patients with complex care needs utilized a smartphone-based remote care management platform and digital health innovations to support self-directed care management [85,97]. While the participant technology comfort level was assessed prerandomization and was expected to be variable among the study population, the study team found that participants often requested assistance in understanding basic phone functionality. Thus, the investigators worked to devise a modality flexible enough to support all levels of technical knowledge, which was easy to access, was user friendly, and addressed a broad range of skills from simple to difficult. The investigators developed a high-tech care strategy user guide with input from the stakeholder partners, patients, and providers. The need for more tailored technical support also resulted in concierge-style in-person technical visits to perform functional assessments that could not be completed over the phone, leading to a new workforce supporting additional technical initiatives across the care delivery continuum.

#### Cultural Tailoring of Telehealth Solutions

When developing telehealth interventions, it is particularly important to consider the need for culturally competent care, defined as care that respects the diverse characteristics and values of the patients, which can ultimately affect their overall health and health care [98]. This review of PCORI-funded studies showed that patient study engagement improved with the use of culturally competent messaging delivered via telehealth interventions.

The MODEL (Management of Diabetes in Everyday Life) study assessed the effectiveness of tailored motivational text messaging for improving diabetes self-care activities in medically underserved African American adults in the Mid-South, and illustrated the importance of cultural tailoring of telehealth solutions [44,45]. This study incorporated feedback from a patient advisory council into the message development process to more competently culturally tailor message content to the target population, an approach that has demonstrated positive impacts in other populations with diabetes [44,45]. The MODEL study’s registry-based text message system employed electronic medical record and survey data, as well as ongoing message input from patients themselves to personalize and deliver culturally tailored content to patients with uncontrolled diabetes [45]. Preliminary program results indicated high satisfaction with the MODEL text message program, with greater than 90% participant retention in this group of the study.

Similarly, the Health-E You/SaludiTu study demonstrated how cultural tailoring of telehealth interventions can improve their acceptance by populations at risk of disparities [91,92]. This study aimed to assess the effectiveness of a patient-centered computer-based clinical intervention to reduce health disparities in unintended pregnancies among Latina adolescent girls in 18 school-based health centers in Los Angeles, California. Latina adolescents and local community representatives were key stakeholders in the design and implementation of this app, which may have helped the adoption and implementation of this intervention into clinic workflows at the school-based health centers. The culturally tailored tool was available in English and Spanish, and adapted language that resonated with Latina adolescents (eg, the use of the more colloquial term *condones* versus *preservativos* to refer to condoms). Preliminary findings showed that both providers and study participants reported high satisfaction with the app, as it was easily integrated into the clinic flow, and more importantly, participants showed an increase in knowledge of contraceptive use from baseline to their 6-month follow-up.

Likewise, early implementation experience from a PCORI-funded study of underserved Asian Americans having chronic hepatitis B suggested that culturally and linguistically competent interventions improve participant retention [72]. This study employed patient-designed culturally tailored text messaging along with patient navigator–led educational sessions to improve adherence to medication therapy for hepatitis B. Patient partners and community stakeholder groups provided extensive input on the appropriateness and framing of the messages. These messages were delivered in Chinese, Vietnamese, or Korean. Early implementation findings showed evidence that this culturally and linguistically appropriate intervention was effective in improving follow-up in patients living with hepatitis B.

#### Delivering Telehealth Through Trusted Intermediaries

Many of the studied PCORI telehealth projects successfully employed trusted intermediaries to deliver telehealth solutions and help support people at high risk for health disparities in crossing the digital divide. These projects provided confirmatory evidence to extensive literature suggesting that lay health worker and paraprofessional intermediaries can help build trust among patients and providers in new telehealth modalities. New technologies, including telehealth, that enable the delivery of interventions and diagnostics remotely are emerging, but patients commonly struggle to use these technologies. Patients struggle with both simple technical issues, such as neglecting to ensure that an electronic system is plugged in, and more complex barriers related to social determinants of health (eg, poverty, unpredictable work schedules, unreliable transportation, and lack of internet access) when using advanced technologies such as secure videoconferencing.

PCORI telehealth projects consistently demonstrate high patient acceptance of telehealth modalities that connect them to trusted providers, but even with careful attention to patient-centered design, investigators commonly found availability of in-person technological support to be essential. For example, when 1 participant in the aforementioned VIGoROUS video gaming intervention reported that her gaming system would not turn on, a support call revealed that she had not plugged it into an electrical outlet [70,71]. This early experience suggested that widescale implementation of telehealth technologies requires funding of responsive technical support infrastructure to avoid technology abandonment by patients in greatest need.

Similarly, a study of Native Americans addressing gaps in self-management through home-based CKD care found that continuous care coordination using trained and trusted tribal community health representatives (CHRs) was essential to effective program implementation [86-88]. This program used videoconferencing with patients via internet-connected portable tablets using Verizon wireless Jetpack mobile hotspots. The program worked with other local health and wellness programs (eg, Indian Health Services and other programs operating within tribal communities) and conducted community-wide health fairs. Telehealth adoption was also encouraged through distribution of culturally sensitive newsletters and brochures to all these health programs and individual community members. The secondary data analysis of the study demonstrated that behavioral and lifestyle educational reinforcement through motivational text messaging and alternate weekly home visits by the CHRs with quarterly group sessions was an effective means of providing care to patients with type 2 diabetes mellitus and CKD who may otherwise avoid diagnosis and treatment due to stigmatization. The CHR-led home-based care model provided the additional care necessary to bolster patient levels of disease-specific knowledge, self-efficacy, and diabetes and CKD self-management, enabling patients to more effectively carry out the recommendations that they received during the home visits, as compared to patients who received clinic-based usual care [99].

A study addressing childhood hearing loss in an Alaska Native population similarly found that using trusted lay health workers based in the communities served was essential to telehealth program deployment [100-103]. In the remote rural communities served by this program, health care is provided by community health aides/practitioners who are commonly from the community they serve and are selected by their community to receive training. This care is supported by specialist triage using the statewide telemedicine network. As a result, ear and hearing problems in isolated communities are routinely managed remotely by audiologists and surgeons through telemedicine, including provision of local care (such as for ear infections), preoperative planning, and postoperative follow-up [102,103]. This requires community health aides/practitioners performing telemedicine consults to be proficient with telemedicine equipment, including basic testing, such as tympanometry, to assess eardrum movement and digital otoscopy for photos of the eardrum. Training with telemedicine equipment is built into each session of the standardized community health aide/practitioner program and includes continued on-the-job training and direct observation of practical skills. Expanding the use of telemedicine equipment for prevention, such as follow-up for referred school hearing screenings, utilizes community health aide/practitioner knowledge and training on the telemedicine infrastructure already in place. However, Emmett et al also provided additional training on the expedited telemedicine consult form to facilitate the application of modified workflows for school hearing screening referrals [68,69].

#### Partnering With Payers to Expand Telehealth Reimbursement

Reimbursement for telehealth encounters is increasing. However, lack of adequate reimbursement continues to present a major challenge to telehealth adoption, implementation, and sustainability. Reimbursement barriers to telehealth were acknowledged by nearly all the PCORI-funded study investigators who participated in this study. Despite recent expansions in telehealth reimbursement during the COVID-19 pandemic, variable state and federal reimbursement policies and low levels of reimbursement for services delivered via telehealth continue to challenge telehealth implementation efforts. In response to these challenges, many studies found it essential to partner with payers to expand their support and/or reimbursement for telehealth services.

For example, the TEAM UP (Treatment Efforts Addressing Child Weight Management by Unifying Patients, Parents, and Providers) study assembled a Payer Advisory Board that explored how military medical sites use telehealth for services such as teleradiology and telebehavioral health [96]. The Board noted how telehealth could facilitate access to qualified and trained health care professionals in rural areas, as well as foster treatment engagement and reduce program attrition by increasing flexibility for patients with challenging schedules. The Payer Advisory Board stressed the viability of telehealth and the need for these types of visits to be considered billable encounters to help overcome the challenges facing populations with barriers to care.

In the rural Alaska study addressing childhood hearing loss, the research team developed a streamlined telemedicine consultation process in order to make it financially sustainable in the school setting [68,69,104]. Translating a clinically oriented technology to a preventive service raises inherent logistical challenges [69]. The team shortened the telemedicine consult time from 60-90 minutes to 10-15 minutes, making it feasible to perform 10 to 15 telemedicine referrals for school hearing screening in a single day. This process took several iterations, with feedback sought from multiple stakeholders including community health aides/practitioners. Maintaining the integrity of billing requirements, while shortening the telemedicine consultation process, allowed the preventive service to remain reimbursable and financially sustainable after the conclusion of the randomized trial.

Similarly, in the Mid-South MODEL study, investigators worked with regional health systems to provide long-term support for a regional registry-based tailored text message system that can assist both large health systems and small independent primary care practices in sending regular motivational messages to interested patients [44,45].

#### Ensuring Confidential Sharing of Private Information

Early implementation experience from numerous PCORI telehealth studies demonstrates the importance of ensuring confidential sharing of private information in all telehealth interventions considered. The application of telehealth often involves paperless collection and transmission of data at each touch point with participants and with communication between staff at the regional and local levels. This requires careful attention to compliance with local, regional, and federal legal requirements for protection of privacy.

For example, the TEAM UP study of alternative childhood obesity treatments for Medicaid-insured individuals employed a secure Health Insurance Portability and Accountability Act (HIPAA)-compliant videoconferencing system to enable remote communication with children and families that protected privacy [96]. Working with a low-income population presents an inherent set of barriers, including unpredictable schedules and unreliable transportation, both of which could negatively impact the fidelity of family-based behavioral treatment delivery. In anticipation of these potential barriers, the study team contracted with a fully HIPAA-compliant videoconferencing system to allow for telehealth delivery of family-based behavioral treatment sessions as needed. Secure video was found to be an ideal technology solution since it is accessible on virtually all devices, including cell phones, and provides necessary flexibility to support adherence with demanding treatment schedules. Use of secure HIPAA-compliant videoconferencing ensured the confidentiality and security of participants’ protected health information in compliance with part 11 of Title 21 of the Code of Federal Regulations pertaining to Electronic Records and Electronic Signatures (21 CFR Part 11) [96]. Likewise, the study employed an electronic consent process that allows for the use of alternative consent forms for each participating site.

The VIGoROUS study also had to address challenges related to information security [70]. First, it was necessary to find a HIPAA-compliant videoconferencing platform for secure video chat between participants and providers. While there are numerous platforms available, some of which are free, the lead site’s hospital system was relatively unfamiliar with telehealth and thus required new technology platforms to undergo an extensive information security vetting process (eg, conducting risk assessments and obtaining administrative approvals), a process that ultimately spanned 3 years. To proceed with the study, investigators were able to identify and pivot to a user-friendly videoconferencing platform (Bluejeans) that had already been vetted by the university health system. The second challenge was how to make large amounts of remote-monitoring data (adherence data, quality of movement data generated continuously during rehabilitation, and behavioral assessment data) accessible to a clinician providing care remotely. This was addressed by streaming deidentified data to a secure cloud server. The data were stored under a unique machine identifier that could only be obtained through physically accessing the rehabilitation gaming system (located within participants’ homes). During video consultations with a therapist, the participant provided the therapist with the machine identifier to enable the therapist to track his/her progress [70].

## Discussion

### Principal Findings

This scoping review of early PCOR evidence suggests that the most effective health system- and provider-level telehealth implementation solutions to address disparities employ patient-centered and culturally tailored telehealth solutions. We found that the development of the most effective telehealth solutions was actively guided by patients themselves in order to meet the needs of specific communities and populations. Early PCOR evidence demonstrates that the best practices in telehealth implementation include delivering telehealth through trusted intermediaries, close partnership with payers to facilitate reimbursement and sustainability, and safeguards to ensure patient-guided confidential sharing of personal health information.

The COVID-19 pandemic has given new urgency to these questions regarding telehealth adoption among people at the highest risk for disparities, including those living in medically underserved, low-income, or rural areas; those from racial and ethnic minorities; LGBT persons; and those with limited English proficiency or disabilities. Telehealth modalities have great potential to help overcome these geographic, socioeconomic, cultural, and language barriers and to give populations at risk of disparities enhanced access to essential health services. Although many PCORI-funded studies had to put their research on hold and transfer their institution’s focus on the needs of patients experiencing COVID-19 [105], the pandemic generally helped to accelerate telehealth adoption for vulnerable populations [26,28]. However, since early evidence indicates that differences in internet and telehealth access may worsen disparities in chronic disease and COVID-19 outcomes [106], it is clear that telehealth solutions must deliberately target and prioritize populations at the highest risk for disparities.

Toward that end, this review places strong emphasis on the importance of deploying patient-centered and culturally tailored telehealth solutions that address the social determinants of health faced by populations at risk for disparities [19-21]. Even though low-income, minority, and rural populations face heightened barriers to effective self-care related to the social determinants of health, most telehealth behavioral interventions for chronic conditions are tailored for individuals with higher incomes. Thus, low-income, minority, and rural groups need specific culturally tailored solutions that specifically address the social determinants of health they face [69,104]. Preliminary evidence from PCORI-funded studies consistently demonstrates that intensive personalization and cultural tailoring of intervention components [70] can help enable vulnerable patients to address critical social determinants of health [71]. Effective telehealth interventions need to address the key social determinants of health at the root of entrenched health behaviors in systematic ways [72]. Most of the reviewed PCOR initiatives employed component interventions that were extensively culturally tailored during the initial program planning phases and on an ongoing basis to ensure that they were culturally congruent and appropriate based on the subjective culture (ie, norms and attitudes), behavioral preferences, and cultural values and expectations of the population served [71,73]. Thus, although cultural tailoring alone is not sufficient, the early PCOR literature indicates that it is an essential component of effective telehealth solutions to address health disparities.

Furthermore, this review revealed that telehealth solutions can take advantage of and expand on existing technological capacities accessible to low-income and other populations at risk for health disparities, and can often be deployed at low cost. The existing PCORI-funded telehealth research suggests that low-cost strategies that employ existing telehealth capacities using standard mobile phones and smartphones may be particularly effective in reaching patients where they are and engaging them in needed care. Although more than 90% of Americans overall carry cell phones and 80% have smartphones [107], among people with household incomes less than US $30,000, only 71% own a smartphone, 54% own some type of computer, and 56% have home broadband [106]. As demonstrated by several of our case studies above, early evidence indicates that text messaging and other culturally tailored mobile health (mHealth) interventions can provide effective low-cost approaches for engaging patients in self-care [108-112]. Some systematic reviews have identified text messaging as among the most effective low-cost technological strategies for engaging patients in behavior change and have highlighted the importance of such technology-supported behavioral interventions [113-115]. Thus, health systems seeking to target telehealth solutions to address disparities should actively seek to employ existing technologies and devices like traditional cell phones to which populations at risk for disparities already have access.

This review also emphasizes the importance of telehealth solutions that work through existing trusted care networks and providers, and that employ trusted lay health worker and paraprofessional intermediaries to introduce and support the use of these technologies by people from the community. Many of the most effective PCORI telehealth initiatives considered in this paper focused their interventions around engaging, training, and deploying trusted intermediaries from the community and/or the population at risk for disparities to be served. This evidence also suggests that personalized human interaction is essential. For example, text messaging interactions were found to be most effective when delivered in real-time, tailored to participant interests and needs, and originated from a trusted known source. The PCORI-funded telehealth projects reviewed suggested that face-to-face videoconferencing and telephone interventions with trusted caregivers are particularly effective.

Our review further suggests that substantive reimbursement and regulatory changes are immediately needed to enable low-cost and efficient telehealth solutions. We found that the best practices in telehealth implementation include close partnership with payers to facilitate reimbursement and sustainability. However, ultimately, experience from the existing portfolio of PCORI telehealth projects indicates that permanent changes are needed in national reimbursement policies and regulations to facilitate broader telehealth adoption and implementation. Policies and reimbursement for telehealth were rapidly put into place to allow health care to continue during the severe restrictions early in the COVID-19 public health emergency after March 2020, in order to allow care to continue during the COVID-19 pandemic. However, these changes clearly need to be extended. Needed regulatory changes include expanded reimbursement as well as relaxations of regulations around where the patient or practitioner is located, licensure, and reciprocity across states. For example, since populations at risk for disparities generally have less access to the internet and technology, regulatory solutions need to facilitate and enable the use of existing lower-cost technologies, such as telephone visits using traditional cell phones, rather than requiring video teleconferencing for medical billing. As the pandemic eases, it will be essential to carefully think about how to preserve the progress that was made in expanding telehealth in ways that improve patient care at the federal (eg, Medicare), state, and individual insurance company levels [116-118].

### Strengths and Limitations

The existing PCORI telehealth research targeting populations at risk for health disparities is of very high quality and has high applicability to real-world clinical and community settings. Of note, 100% of the PCORI-funded studies in this category employed a randomized controlled trial design and 100% were pragmatic rather than being conducted in highly controlled university settings among highly selected patients. This is a major strength of both the research reviewed in this scoping review and the scoping review itself. Furthermore, the studies included in this scoping review are also notable for their generalizability, given their pragmatic real-world approach. Rather than excluding patients with multiple chronic conditions as is often done in randomized clinical trials, many of the PCORI-funded studies specifically included patients with multiple morbidities given their representativeness of patients most commonly seen in real-world clinical settings.

This review is limited by the small numbers of studies that were considered, and its main findings are subject to numerous potential biases. First, since PCORI staff chose studies for an in-depth review based on their perception regarding which study teams overcame notable obstacles to telehealth, selection bias could have occurred. Our expectation is that this bias would most likely lead to the overemphasis of facilitators and solutions as opposed to barriers and challenges. Second, because PCORI staff and investigators comprised the study team, it is possible that there was some bias toward reporting of positive study findings. However, the study team and methods actively sought to address this potential limitation by identifying and categorizing barriers first prior to the discussion of solutions. In addition, some of the telehealth services provided in these studies may be difficult to consistently replicate outside of structured evaluations.

The review is also limited because final study results are not available for many of the included studies, and even fewer have detailed analyses of implementation factors and lessons learned available in the peer-reviewed literature. However, the review was facilitated by the detailed project knowledge of PCORI staff and the investigators themselves.

### Future Directions

Over the past 5 years, PCORI has supported many comparative effectiveness studies of adopting virtual care solutions to manage health outcomes across a wide range of populations at risk for disparities. Through these research initiatives, health care delivery systems are learning to leverage low-cost telehealth solutions to promote better health outcomes by increasing access to care, making care more effective, and continuously engaging patients. PCORI-funded comparative effectiveness research is demonstrating how we can reduce health disparities across the spectrum of disease conditions and populations through low-cost telehealth solutions. This research is identifying key barriers and limitations to implementing telehealth in vulnerable populations and ways to overcome these barriers to help improve health and health care outcomes.

In this new era of the COVID-19 pandemic, there has been rapid growth in the use of technology across all industries, but it is proving to be particularly important in the areas of health and health care. Future efforts should focus on fostering collaborations between researchers, payers, and administrators to facilitate widespread adoption of new treatment models that have already demonstrated success through definitive clinical trials. Our review indicates that key system- and policy-level changes are desperately needed to address barriers to telehealth reimbursement. Moreover, further pragmatic research is needed to demonstrate the best approaches for disseminating and scaling these early telehealth findings in broader community practice to promote population health.

### Conclusions

This scoping review gives strong guidance to health systems seeking to target evidence-based telehealth modalities for health disparities. Results from the studies demonstrate that systems can do so in innovative ways, and in fact, some of the studies identify methods for systems to effectively reach underserved populations in their own communities. These case studies also highlight the critical importance of both supportive infrastructure, and regulatory and reimbursement policies to facilitate telehealth modalities and make them sustainable.

However, despite growing evidence that telehealth can deliver care to people at risk for disparities, many major obstacles exist for its implementation. Early PCOR evidence demonstrates that the most effective health system- and provider-level solutions for at-risk populations use patient-centered and culturally tailored telehealth solutions whose development is actively guided by the patients themselves.

## Appendix

Multimedia Appendix 1 Patient-Centered Outcomes Research Institute–funded studies assessing the use of telehealth to improve outcomes for populations at risk for health or health care disparities.

## Abbreviations

CHR: community health representative
CKD: chronic kidney disease
HIPAA: Health Insurance Portability and Accountability Act
LGBT: lesbian, gay, bisexual, and transgender
MODEL: Management of Diabetes In Everyday Life
PCOR: patient-centered outcomes research
PCORI: Patient-Centered Outcomes Research Institute
PRISMA: Preferred Reporting Items for Systematic Reviews and Meta-Analyses
TEAM UP: Treatment Efforts Addressing Child Weight Management by Unifying Patients, Parents, and Providers
VIGoROUS: Video Game Rehabilitation for Outpatient Stroke
