# Supplementary material for: Early Patient-Centered Outcomes Research Experience With the Use of Telehealth to Address Disparities: Scoping Review
Source: J Med Internet Res. 2021 Dec 7;23(12):e28503. doi: 10.2196/28503 (PMC8693194; doi:10.2196/28503)
Supplement: Multimedia Appendix 1 [file jmir_v23i12e28503_app1.docx]

**Multimedia Appendix 1**. Patient-Centered Outcomes Research Institute–funded studies assessing the use of telehealth to improve outcomes for populations at risk for health or health care disparities.

| **Principal Investigator, Organization, and Project Title** | **Primary Study Goal** | **Telehealth Intervention** | **Setting** | **Completed (or Planned) Participants**  **(No.)** | **Targeted Study Population** |
| --- | --- | --- | --- | --- | --- |
| Armstrong AW, University of Southern California,  Improving specialty care delivery in chronic skin care [42, 43] | To evaluate whether connecting patients and primary care physicians with dermatologists online (Collaborative Connected Health) is as effective as sending patients to an in-person appointment with a dermatologist. | Online visits with derma-tologists | Multicenter ambulatory clinics in Northern California, Southern California, and Colorado | 296 (online Collaborative Connected Health [n=148] vs. usual in-person psoriasis care [n=148]) | Adults with psoriasis |
| Bailey, JE, University of Tennessee Health Science Center,  Improving Self-Care Decisions of Medically Underserved African Americans with Uncontrolled Diabetes: Effectiveness of Patient-Driven Text Messaging versus Health Coaching [44, 45] | To compare how well certain approaches (motivational text messaging vs. health coaching vs. enhanced usual care) help African American people with diabetes improve their self-care. Types of self-care include healthy eating, physical activity, and medication adherence. | Patient-driven (tailored) motivational text messages from the doctor’s office | Mid-South primary and specialty care practices in medically underserved areas of Tennessee, Mississippi, and Arkansas | 646 (text messaging [n=258] vs. health coaching [n=258] vs. enhanced usual care [n=130]) | African American adults with uncontrolled diabetes and multiple chronic conditions |
| Blalock SJ, The University of North Carolina at Chapel Hill, Enhancing patient ability to understand and utilize complex information concerning medication self-management [46] | To compare the effectiveness of DrugFactsBoxes© delivered vs. Medication Guides mandated by the FDA (both with and without Gist Reasoning Training [SMART]) in patients with rheumatoid arthritis (RA) on informed decision making. | Online patient support community | Large academic  rheumato-logy practices and general population in North Carolina | 312 (Medication guides [n=80] vs. Medication guides+SMART [n=79] vs. DrugFactBoxes [n=74] vs. DrugFactBoxes+SMART [n=76]) | Adults with moderate or highly active rheumatoid arthritis |
| Bossarte R, West Virginia University Robert C. Byrd Health Sciences Center, Remote Cognitive Behavior Therapy for Major Depression (RTD) in Primary Care [47] | To compare the effectiveness of eCBT  plus treatment as usual (TAU) vs. unguided remote internet-based CBT plus TAU vs. TAU alone in patients with major depressive disorder (MDD). | Remote internet-based cognitive behavior therapy (eCBT) | Federally qualified health centers (FQHC) in rural West Virginia | 8,000 (usual care [n=2,667] vs. usual care plus remote unguided eCBT [n=2,667] vs. usual care plus remote guided eCBT [n=2,667]) | Adults seeking outpatient treatment for major depressive disorder for the first time in the past 6 months |
| Bowen C, Regents of the University of California, Los Angeles, Resilience Against Depression Disparities  [48] | To compare the effectiveness of providing evidence-based depression care training and toolkits vs. provision of training and toolkits plus a tailored Cognitive Behavioral Therapy (CBT)-informed intervention to improve depression. | Webinars, text messages and follow-up care reminders | Primary care, mental health, and community agencies that predominately serve lesbian, gay, bisexual, transgender, and queer (LGBTQ) patients in Los Angeles, California and New Orleans, Louisiana | 265 (RS+ Resiliency Class group [n=127] vs. Resiliency only group [n=126]) | African American and/or Latino LGBTQ adults with moderate to severe depression |
| Coker T,  Seattle Children's Research Institute, Using Telehealth to Deliver Developmental, Behavioral, and Mental Health Services in Primary Care Settings for Children in Underserved Areas [49, 50] | To compare the effectiveness of video chat referral to the usual complex referral process in helping families with children who have Medicaid get needed mental health care. | Video chat referrals for mental health care | Six health centers in Los Angeles County,  California | 359  (telehealth-based referral process [n=164] vs. usual referral process [n=178]) | Parents of children 5-12 years old with Medicaid in underserved areas referred for mental health care |
| Cook J, Yale University, Peer Online Motivational Interviewing for Sexual and Gender Minority Male Survivors [51] | To compare the effectiveness of motivational interviewing to motivational interviewing with trauma-informed sexual and gender minority affirmative care. | Peer-led online group counseling | Nationwide sample | 344 (Motivational interviewing [n=172] vs. Motivational Interviewing with trauma informed sexual and gender minority affirmative care [n=172]) | Sexual and gender minority men with a history of sexual abuse and high emotional distress |
| Critchfield A and Hansen W, University of Kentucky Research Foundation, The PATH Home Trial: A Comparative Effectiveness Study of Peripartum Opioid Use Disorder in Rural Kentucky [52] | To compare the effectiveness of nurse- and specialist-led peer support group sessions with telemedicine visits with specialists in substance use and in mother and child health on incidence of neonatal abstinence syndrome requiring treatment. | Telemedicine visits with substance abuse and maternal-infant health specialists | Twelve prenatal clinics in rural Kentucky | 1620 (Group sessions [n=810] vs. Telemedicine [n=810]) | Pregnant women in rural Kentucky receiving medication-assisted therapy for opioid use disorder |
| Dehlendorf CE,  University of California, San Francisco,  Patient-Centered Support for Contraceptive Decision Making [53] | To compare use of contraceptive counseling with the My Birth Control decision aid against contraceptive counseling alone. | Decision-aid tool on a tablet | Four health clinics in San Francisco, California | 758 (Contraceptive counseling with decision aid [n=396] vs. Usual Care [n=348] (14 total did not receive their allocation) | Low-income English- and Spanish-speaking women  wishing to start or change contra-ceptive methods |
| Dhar, Northwestern University, Ear, Nose, and Throat Diseases  Hearing Loss [54] | To compare two over-the-counter models of hearing aid distribution and fitting (Brief Consumer Decides Model [bCD] and Efficient Fit Model [EF]) against audiology-based best practice (AB), with and without supplemental video learning modules. | Video-based supplemental education program | Four research sites in Chicago, IL and Galveston, TX | 306 (AB+ [n=51]) vs. bCD+ [ n=51] + [n=51] vs. AB- [n=51]] vs. bCD- [n=51] vs. EF- [n=51] | Older adults with mild or moderate hearing difficulty |
| Dorsey RE,  University of Rochester, Using technology to deliver multi-disciplinary care to individuals with Parkinson disease in their homes [55-57] | To learn if video house calls with specialists were convenient and improved quality of life for people with Parkinson’s disease. The team also wanted to learn if video house calls improved quality of care, reduced travel time, and reduced burden on caregivers due to the demands of care. | Video house calls with specialists | 20 centers serving underserved counties, Multi-state | 195 (and 111 caregivers) (Video house calls [n=97]; (50 caregivers) vs. Usual Care [n=98](61 caregivers)) | Adults with Parkinson’s Disease in medically under-served areas, not currently seeing a neurologist |
| Fiscella KA, University of Rochester, Addressing HIV Treatment Disparities using a Self-Management Program and Interactive Personal Health Record [58, 59] | To assess the effectiveness of a multicomponent program (including an iPod app, group-based training, a meeting with a health coach, and training for doctors [GREAT intervention]) for patients with HIV to improve skills for managing their health. | Web-enabled hand-held device (Apple iPod Touch) loaded with a Personal Health Record (ePHR) customized for HIV patients | Eight clinics in New York and New Jersey | 360 (GREAT [n=180] vs. standard clinical care [n=180]) | Adults with confirmed HIV diagnoses |
| Fortney JC,  University of Washington, Integrated Versus Referral Care for Complex Psychiatric Disorders in Rural FQHCs [60, 61] | To compare two telemedicine options (telepsychiatry-collaborative care [TCC] and telepsychiatry-enhanced referral [TER]) for patients with post-traumatic stress disorder (PTSD) and bipolar disorder. | Telepsychiatry-collaborative care vs. telepsychiatry-enhanced referral | 15 rural community health centers in Arkansas,  Michigan, and  Washington | 1,000 (TCC [n=500] vs. TER [n=500]) | Adults in rural areas who screen positive for PTSD and/or bipolar disorder |
| Fortuna, LR,  Boston Medical Center Corporation,  KIDS FACE FEARS: Face-to-face vs. Computer-Enhanced Formats Pragmatic Study of Anxiety [62] | To compare the effectiveness of face-to-face (f2f) versus online treatment (Cool Kids Online CBT) for youth anxiety, evaluate both acute and longer-term outcomes across a two-year follow-up period, and test whether the relative effectiveness of each of these two treatment formats varies for different patient subpopulations or circumstances. | Online CBT | Pediatric health settings, Multistate | 3,140 (Cool Kids f2f CBT [n=1,570] vs. Cool Kids Online CBT [n=1,570]) | Youth ages 3 to 18 with anxiety disorders |
| Hajizadeh N,  Feinstein Institute for Medical Research, A comprehensive disease management program to improve quality of life in disparity Hispanic patients admitted with exacerbation of chronic pulmonary diseases [63, 64] | To compare the effectiveness of telehealth pulmonary rehabilitation led by a respiratory therapist (TelePR) and standard pulmonary rehabilitation at an outpatient clinic (SPR) in reducing hospitalization and improving health. | Telehealth COPD therapy | New York City area hospitals and outpatient clinics | 176 (Referral to TelePR [n=88] vs. Referral to SPR (usual care) [n=88]) | African American and Latino adults with chronic obstructive pulmonary disease (COPD) from underserved communities recently discharged from the hospital |
| Halpern, University of Pennsylvania, Comparing Smoking Cessation Interventions among Underserved Patients Referred for Lung Cancer Screening [65] | To compare four programs/ways to help patients quit smoking. | Ecological momentary intervention (EMI) mHealth app | Four diverse health systems across the U.S. | 3,200 [Ask-Advise-Refer [n=800] vs. Ask-Advise-Refer + free prescription medicine [n=800] vs. Advise-Refer + free prescription medicine + EMI mHealth [n=800] vs. all approaches [n=800]) | Current smokers recommended for lung cancer screening who are Black or Hispanic and/or low-income or live in a rural area |
| Jonassaint C and Abebe K, University of Pittsburgh, Cognitive Behavioral Therapy and Real-Time Pain Management Intervention for Sickle Cell via Mobile Applications (CaRISMA) [66] | To compare the effectiveness of telemedicine-delivered cognitive behavioral therapy (mCBT) and pain education (mEd) to improve pain outcomes for adults with sickle cell disease. | mCBT and mEd delivered via a smartphone application | Clinics and community-based organizations | 350 (mCBT for pain [n=175] vs. mEd [n=175]) | Adults with sickle cell disease who report chronic pain |
| Katz RJ, George Washington University,  Changing the Healthcare Delivery Model: A Community Health Worker/Mobile Chronic Care Team Strategy [67] | To compare the effectiveness of three ways to help patients manage their diabetes: daily cell phone text message reminders (C4L), talking with a community health worker with diabetes trained to help others with diabetes get the health care they need (CHW), and both text message reminders and talking to a community health worker (C4L+CHW). | Interactive text message reminders (C4L) | Three clinics in the Washington, DC area | 166 (C4L [n=56] vs. CHW [n=56] vs, C4L+CHW [n=54]) | Predominantly African American adults with Medicaid and type 2 diabetes |
| Kleindienst Robler S and Emmett S,  Norton Sound Health Corporation, Addressing Childhood Hearing Loss Disparities in an Alaska Native Population: A Community Randomized Trial [68, 69] | To determine the comparative effectiveness of mHealth screening and a novel telemedicine specialty referral pathway compared to standard screening and a standard primary care referral pathway in identifying children with hearing loss and improving their outcomes. | mHealth screening, telemedicine specialty referral intervention | 15 majority Alaska Native communities in remote rural areas of Northwest Alaska | 1,219  (Telemedicine [n=617] vs. usual care [n=602]) | Predominantly Alaska Native children ages 4–21 attending school in remote rural areas |
| Larsen D and  Gauthier LV,  The Ohio State University,  Comparative Effectiveness of a Low-Cost Virtual Reality Gaming Platform for Neurorehabilitation of Hemiparesis [70, 71] | To compare the effectiveness of in-person constraint-induced movement therapy (CI), CI + video game therapy (Gaming), CI + Gaming + Telehealth monitoring (TM), and traditional in-person physical therapy in improving hand function in people with past strokes (Standard care) | Online video avatar-based virtual reality game CI therapy, videoconference sessions with physical therapist | Four medical facilities in Alabama, Ohio, and  Oregon | 193 (CI [n=50] vs. CI + Gaming [49] vs. CI + Gaming + TM [n=50] vs. Standard Care [n=44]) | Adults with a stroke at least six months prior resulting in mild to moderate hemiparesis |
| Ma GX, Temple University,  A Comparative Trial of Improving Care for Underserved Asian Americans Infected with HBV [72] | To assess the effectiveness of a multicomponent program including in-person sessions, web-based educational programs, text message reminders, and patient navigator (PN) support in improving receipt of recommended care for chronic hepatitis B (CHB) and patient outcomes. | Text message follow-up care reminders and web-based educational programs | Pennsylvania,  New York, and  New Jersey | 532 (PN-led CHB management education [n=272;] vs. usual care [n=260]) | Asian-American adults living with chronic hepatitis B infection |
| Marcin J, The Regents of the University of California, Davis, Improving Family-Centered Pediatric Trauma Care -- The Standard of Care versus the Virtual Pediatric Trauma Center [73] | To compare current standard of pediatric trauma care to Virtual Pediatric Trauma Center model of care. | Virtual Pediatric Trauma Center (VPTC) live video | Ten hospitals throughout rural northern California | 380 (VPTC [n=190] vs. Usual Care [n=190]) | Pediatric trauma patients |
| Margolis KL, HealthPartners Institute, Pragmatic Trial Comparing Telehealth Care and Optimized Clinic-Based Care for Uncontrolled High Blood Pressure [74] | To compare the effectiveness of two ways (ie, face-to-face with doctors and medical assistants to discuss and track blood pressure vs. home-based care by a pharmacist or nurse practitioner to track blood pressure using a home blood pressure monitoring device) for healthcare teams to help patients lower their systolic blood pressure and improve health outcomes. | Home-based telehealth care | 21 primary care clinics in an integrated health system in Minnesota and Wisconsin with Medication Therapy Management  pharmacists | 3,072 (Clinic-based care [n=1,648] vs. Telehealth [n=1,424]) | Adults with uncontrolled hypertension |
| Osunkwo I,  Lawrence R; Carolinas Medical Center; Comparative Effectiveness Of Peer Mentoring Versus Structured Education Based Transition Programming For The Management Of Care Transitions In Emerging Adults With Sickle Cell Disease (SCD) [75] | To compare the effectiveness of two ways for pediatric and adult clinics to help patients with transition successfully to adult care—structured education-based program (STE) vs. STE plus a one-on-one, virtual peer mentoring program (PM)—in improving quality of life and decreasing emergency room visits. | Virtual peer mentoring program (PM) | 14 sites with a pediatric SCD clinic, an adult SCD clinic, and a partner community-based organization  in 8 states (North Carolina, South Carolina, Georgia, Kentucky, Alabama, Mississippi, and Virginia) | 700 (STE+PM [n=350] vs. STE [n=350]) | Teens and young adults ages 16 to 25 with SCD |
| Page K, John Hopkins School of Medicine, Leveraging mHealth and Peers to Engage African Americans and Latinos in HIV Care [76] | To compare the effectiveness of a Linkage to Care (LTCR) program that connects African American and Latino adults with HIV to care by making same-day appointments and providing transportation to clinic with and without a smartphone application (mHealth enhanced LTCR) that assists patients in getting needed care. | A Linkage to Care smartphone application that helps make appoint-ments, sends text messages and appointment reminders, refills medicines, answers questions, and alerts the care team for lab results. | Baltimore City Health Department, Baltimore, Maryland | 500 (LTCR [n=250] vs. mHealth-enhanced LTCR services [n=250]) | Adults with a new or existing HIV diagnosis |
| Pekmezaris R,  Feinstein Institute for Medical Research, Telehealth Self-Management Program in Older adults Living with Heart Failure in Health Disparity Communities [77, 78] | Comparing the effectiveness of telehealth self-monitoring and nurse video calls vs. routine outpatient management in decreasing emergency visits and hospitalizations and improving quality of life. | Remote patient monitoring with a computer system, weekly video calls with a nurse | A hospital in New York serving people with low income | 104 (telehealth self-monitoring [n=46] vs. clinic outpatient management [n=58]) | Black and Hispanic adults recently hospitalized with heart failure |
| Pekmezaris R,  Feinstein Institute for Medical Research,  Patient and Caregiver-Centered Diabetes Telemanagement Program for Hispanic/Latino Patients [79] | Comparing the effectiveness of diabetes telemanagement (DTM) and usual care alone comprehensive in improving diabetes control and patient-centered outcomes. | Diabetes telemanagement (DTM) including remote telemonitoring and virtual visits for diabetes care and education | New York City area | 240 (Usual diabetes care plus DTM [n=120] vs. usual diabetes care alone [n=120]) | Hispanic adults diagnosed with type 2 diabetes |
| Rosas LG, Stanford University School of Medicine,  The HOMBRE Trial: Comparing Two Innovative Approaches to Reduce Chronic Disease Risk among Latino Men [80] | To assess the effectiveness of a flexible package of a culturally tailored and health coach-delivered behavioral interventions in maintaining clinically significant weight loss (≥5 percent) at 18 months. | Online virtual groups | A large primary care system in  California | 424 (HOMBRE [n=212] vs. minimal intensity control [n=212]) | Adult Latino men with obesity and ≥1 metabolic syndrome component |
| Rosenberg L, Zhou E; Trustees of Boston University, BUMC  Reducing Health Disparities for Black Women in the Treatment of Insomnia [81] | To compare the effectiveness of three treatments for insomnia—an online CBT program (SHUTi), an online CBT program tailored for Black women (SHUTi-BW), and usual care—among Black women. | Online CBT program (SHUTi and SHUTi-BW) | Nationwide sample | 303 (SHUTi [n=101] vs. SHUTi-BW [n=101] vs. usual care [n=101]) | Black women ages 44 to 93 participating in the Black Women’s Health Study with clinically elevated symptoms of insomnia |
| Rothman R, Vanderbilt University Medical Center, Greenlight Plus Study: A Randomized Study of Approaches to Early Childhood Obesity Prevention [82] | Comparing two ways to promote health weight gain and help prevent obesity in infants, including educational materials (Greenlight) vs. access to an educational website and regular text messages (Greenlight Plus).  . | Access to an educational website and regular text messages to parents on setting and reaching behavior goals that promote healthy weight gain for babies (Greenlight Plus) | Six primary care clinics in Tennessee | 900 (Greenlight [n=450] vs. Greenlight Plus [n=450]) | English-and-Spanish-speaking parent-infant pairs |
| Rubin DM, The Children’s' Hospital of Philadelphia,  Community Health Workers and Mobile Health for Emerging Adults Transitioning Sickle Cell Disease (SCD) Care (COMETS Trial)  [83] | To see if community health workers (CHW) or a smartphone application (iManage), when compared with usual care, can improve patients’ quality of life, increase patients’ ability to manage their SCD on their own, and reduce how often patients go to the hospital. | iManage mobile health application with tailored text messaging | Five children’s hospitals in four US cities in Pennsylvania and  Ohio | 450 (CHW [n=150] vs. iManage [n=150] vs. Usual Care [150]) | Young adults ages 17 to 24 with SCD |
| Sadasivam R, University of Massachusetts Medical School,  Smoker-to-Smoker Peer Marketing and Messaging to Disseminate Tobacco Interventions [84] | To test website features that encourage people to sign up for Decide2Quit. The team also wants to compare two computer programs that give personalized messages about quitting smoking on the Decide2Quit website. | Tailored text messaging | Multi-state | 1,463 (Fully enhanced [n=371] vs. Recommender system only [n=370] vs. Peer Recruitment Tools Only [n=374] vs. Standard Care [n=372]) | Current smokers ages 18 and older who can read or speak English and have internet access at home |
| James M. Schuster, MD, MBA  UPMC Center for High-Value Health Care  Leveraging Integrated Models of Care to Improve Patient-Centered Outcomes for Publicly Insured Adults with Complex Health Care Needs [85] | To compare three ways (High-Touch care strategy, High-Tech care strategy, Usual care strategy) to help patients who have two or more chronic conditions manage their health and health care after a hospital stay. | Self-management tool plus virtual visits with a care manager | Pennsyl-vania | 1,662 (High-Touch [n=667] vs. High-Tech [n=667] vs. Usual Care [n=328]) | Adults 21yo or older who qualify for Medicaid or who are dual (Medicare-Medicaid) eligible and who have multiple comorbidities, polypharmacy, and/or risk of high future healthcare utilization |
| Vallabh O. Shah, PhD  University of New Mexico Health Sciences Center  Reducing health disparity in chronic kidney disease in Zuni Indians [86, 87] | To learn if an at-home education program would help Zuni adults with kidney disease have better knowledge, skills, and confidence to manage their illness. The team also looked at whether the program helped Zuni adults improve their quality of life and reduce risk factors for kidney disease. | Home-based Kidney Care (HBKC) videoconferencing through tablets | New Mexico | 125 (HBKC [n=63] vs. Usual Care [n=62]) | Adult Zuni Indians living with chronic kidney disease (CKD) |
| Vallabh O. Shah, PhD  University of New Mexico Health Sciences Center  Home-Based Chronic Kidney Disease (CKD) Care in Native Americans of New Mexico – A Disruptive Innovation [88] | To know if culturally tailored at-home education delivered by community health workers can help people with CKD in four Native American communities feel more confident about managing their CKD and can help them take steps that slow its progress. | Home-based Kidney Care (HKBC), videoconferencing through tablets | New Mexico | 206 (HKBC [n=103] vs. delayed group intervention [n=103]) | Adults ages 21 to 80 who have diabetes or HbA1c>7 and BMI >27 kg/m2 |
| Brian Skotko, MD, MPP  Massachusetts General Hospital  Virtual Evidence-based Healthcare for Underserved Patients with Down Syndrome [89] | To test a website that creates personalized reports for families of patients with Down Syndrome and letters for their doctors to help patients get recommended screening tests. | Web-based platform called Down Syndrome Clinic to You (DSC2U) with personalized reports | Multi-state | 230 (DSC2U [n=117] vs. Usual Care [n=113]) | Children 18 and under, individuals with disabilities |
| Stone K, Sutter Bay Hospitals, Comparative Effectiveness of Zolpidem and Cognitive Behavioral Therapy for Insomnia (CBT-I) in Rural Adults (COZi-R) [90] | To compare the effectiveness of zolpidem, CBT-I, and combination treatment for insomnia symptoms. | Internet CBT-I | 8 healthcare systems across the U.S. | 1,200 (zolpidem [n=400]) vs. Internet CBT-I [n=400] vs. zolpidem + Internet CBT-I [n=400]) | Adults with insomnia severity index score > 10 |
| Kathleen Tebb, PhD  University of California, San Francisco  Reducing Health Disparities in Unintended Pregnancies Among Hispanic Adolescents Using a Patient-Centered Computer-Based Clinic Intervention  [91, 92] | To test ways to help Latina teens in 18 school-based health centers (SBHCs) learn about birth control methods and choose a method that works well for them. | Smart phone application (Health-E You/Salud iTu) | California | 1354 (Health-E You/Salud iTU app [n=693] vs. Usual Care [n=667]) | Latina adoles-cents ages 14 to 18 who are at risk of becoming pregnant or getting sexually transmit-ted infections |
| Van Voorhees B, University of Illinois at Chicago, Primary Care and Community-Based Prevention of Mental Disorders in Adolescents [93] | To compare two depression prevention programs including a self-guided online program (CATCH-IT) and a face-to-face group program with other teens called Teens Achieving Mastery Over Stress (TEAMS). | Self-guided online program (CATCH-IT) | Chicago, Illinois and Louisville, Kentucky | 564 (TEAMS [n=282] vs. CATCH-IT [n=282]) | Inner-city, suburban, and rural communities of African American, Latino, Arab, and White teens with minor symptoms of depression |
| David Wetter, PhD  University of Utah  Multi-Level Interventions for Increasing Tobacco Cessation at Federally Qualified Health Centers (FQHCs)  [94, 95] | To increase the reach and impact of evidence-based tobacco cessation treatment (clinic-level intervention that focuses on enhanced system supports at point of care using the electronic health record (EHR); and two patient-level interventions that both increase opportunities to engage in Quitline treatment and provide motivation and practical problem-solving strategies for addressing barriers to quitting and treatment engagement. | Monthly text messages (TM) plus phone calls with a health educator | 33 primary care clinics within 11 FQHCs in multiple states | 6,000 (5,100 randomized to TM [n=4080] vs. Continued EHR intervention [n=1,020]; 3,264 randomized to TM + Motivation and Problem Solving [n=1632] vs. Continued TM [n=1,632]) | Current tobacco users ages 18 and older |
| Denise Wilfley, PhD  Stephen Cook, MD, MPH  Washington University  A Pragmatic Family Centered Approach to Childhood Obesity Treatment [96] | To compare two treatment models, enhanced standard of care (eSOC) plus family-based behavioral treatment (FBT) vs. eSOC, to provide families and PCPs with information on the best intervention approach for the behavioral treatment of childhood obesity. | Telehealth FBT | Missouri  New York  Louisiana | 1296 child/parent dyads (eSOC [n=648] vs. eSOC + FBT [n=648]) | Children ages 6-15 years of age with a BMI greater than the 95^th^ percentile |
